# The role of immunity and inflammation in obsessive-compulsive disorder and related conditions

**DOI:** 10.1192/j.eurpsy.2024.1305

**Published:** 2024-08-27

**Authors:** S. Palermo, R. Gurrieri, E. Annuzzi, M. Gambini, A. Arone, A. Coccoglioniti, G. D’Angelo, D. Marazziti

**Affiliations:** ^1^Department of Clinical and Experimental Medicine; ^2^Department of Surgical-Medical and Molecular Pathology and Critical Care Medicine, University of Pisa, Pisa; ^3^Saint Camillus International University of Health and Medical Sciences, UniCamillus, Rome, Italy

## Abstract

**Introduction:**

In recent years, there has been a growing interest in the potential consequences of disruptions in the inflammatory and immune systems on the onset of obsessive-compulsive disorder (OCD) and associated conditions, such as Paediatric Autoimmune Neuropsychiatric Disorders Associated with Streptococcal Infection (PANDAS) and Tourette syndrome (TS). While this area of inquiry is undeniably captivating, the available data remain somewhat controversial and limited in scope.

**Objectives:**

The aim of this paper is to conduct an exhaustive examination and evaluation of the existing body of literature concerning aberrations in inflammatory and immune system processes within the context of OCD, PANDAS, and TS.

**Methods:**

This narrative review entailed a comprehensive search of English language papers on PubMed and Google Scholar from January 1985 to July 31, 2023.

**Results:**

The data collected up to this point suggest that the underlying mechanisms at play may differ depending on the age of the patients and the specific disorder being investigated. Notably, PANDAS seems to have a stronger connection with infections that trigger autoimmunity, which may not necessarily be limited to those resulting from Group A beta-haemolytic streptococcal (GABHS) infections, as previously assumed. In the case of TS, autoimmunity appears to play a significant role, especially when combined with individual susceptibilities stemming from both genetic and environmental factors. As for adult OCD, while the available data are somewhat scattered and occasionally based on relatively small groups of patients, they do indicate that the immune system and inflammatory processes are involved in the disorder’s pathophysiology. However, it remains uncertain whether these processes are primary driving forces or secondary reactions.

**Conclusions:**

In summary, when viewed collectively, the current research findings unveil promising avenues for exploring the underlying causes of OCD and related disorders. They also hold the potential for the development of innovative therapeutic approaches that go beyond the current pharmacological paradigms.

**Disclosure of Interest:**

None Declared

